# Durability Performance Indices for Cement-Based Mortars

**DOI:** 10.3390/ma14112758

**Published:** 2021-05-23

**Authors:** Rebeca Visairo-Méndez, Andrés A. Torres-Acosta, Roberto Alvarado-Cárdenas

**Affiliations:** 1 Departamento de Posgrado, Facultad de Ingeniería, Universidad Autónoma de Querétaro, Santiago de Querétaro 76117, Mexico; rvisairom@gmail.com (R.V.-M.); ralvaradocardenas@gmail.com (R.A.-C.); 2 School of Engineering and Science, Tecnologico de Monterrey, Santiago de Querétaro 76130, Mexico

**Keywords:** durability, electrical resistivity, empirical correlations, rapid chloride permeability, repair mortar

## Abstract

Corrosion-induced damaged structures are generally repaired using locally available materials. Nevertheless, determining the durability of the repair materials to be used is necessary to forecast its service life after being placed on the damaged structure. In previous investigations, the most commonly used durability indices are saturated electrical resistivity (*ρ*_S_), ultrasonic pulse velocity (UPV), total void content (TVC), water capillary absorption (WCA), rapid chloride permeability (RCP), and compressive strength (*fc*). Four repair mortar types were evaluated. For each mortar type, 5 × 5 cm^2^ cubes, 5 × 10 cm^2^ (small) cylinders, and 10 × 20 cm^2^ (large) cylinders were made from each mortar evaluated. On the basis of the present results, the durability design of mortars should consider not only the mechanical strength, but also the durability index values to define its durability performance. According to the empirical correlations obtained between all durability indices, *ρ*_S_ vs. RCP, TVC vs. WCA, and RCP vs. WCA were the ones with higher correlation coefficient. These correlations could be used for mortar mixture durability forecasting.

## 1. Introduction

Concrete is a cost-effective and readily available construction material. It has various properties and attractive characteristics, making it convenient for numerous construction applications. For example, for modern structures, the workability of concrete allows forms that require precise designs [1]. Concrete can be fluid in such a way that even 3D prints can be made for experimental models or structural elements in service; this requires an excellent mix design and quality control of materials [2].

In previous investigations, it has been emphasized how concrete durability begins with the design of structural elements from durability criteria [3,4]. By considering first durability design, it has been shown that the mechanical strength of the structure obtained is much greater than the selected from just mechanical strength design. This is because the demands on the materials’ properties against harsh environments go beyond the mechanical performance of the structural element. Once the durability design of the structural elements is achieved, the mechanical performance follows [3].

Concrete structures can be durable, but can be damaged from the environment to which they are exposed. The hydrated products of the cement paste passivate the steel surface. However, the pores formed during the hardening of the concrete sometimes allow aggressive ions (e.g., Cl^−^) to accumulate on the steel surface and break the passive film, thus initiating oxidation–reduction reactions [5].

Diverse methods have been developed to avoid such damage and extend the life and service of such structures by minimizing the damage and corrosion of the reinforcing steel. Such techniques can be applied to concrete (e.g., porosity reduction, concrete cover thickness increase, and corrosion inhibitors) or the reinforcing steel (e.g., epoxy or metallic coatings, corrosion-resistant reinforcement such as stainless steel, and cathodic protection) [6,7,8,9]. However, to actual in-service structures, in which such durability designs are not considered, environmental aggressiveness may cause visible damage that could compromise their load capacity in a short period (<10 years) [10,11,12].

Once a structure is corroded and considerable damage appears on the concrete surface (or in the reinforcing steel), repairing the structure becomes necessary before damage propagates to its other zones. In several cases where the load capacity and the concrete section have been decreased because of this corrosion degradation, replacing the steel reinforcement or using corrosion-free reinforcing materials such as carbon fiber composites is necessary [13,14].

In structures in which corrosion has not yet considerably affected the reinforcing steel (i.e., <5% of rebar diameter loss), corrosion repair could be performed using the well-known patching repair method. In this repair method, a mortar or concrete is placed in areas where corrosion has resulted in cracks or concrete cover delamination [14,15,16].

The patch repair method includes the following steps:

1. Delimitation of the surface to be repaired.
2. Demolition of the damaged concrete delimited previously to completely uncover the steel reinforcement (at a depth of up to 2–3 cm behind the reinforcement).
3. Surface cleaning/preparation where the new repair material will be placed (mortar or concrete).
4. Adherence bridge application, which would adequately bond the repair material (mortar or concrete) to the old concrete.
5. Repair material application (mortar or concrete) according to the thixotropic properties of the repair material.
6. Repair material curing, protection against external contamination, direct solar radiation, and other relevant steps.

Each of these steps should be followed and scrutinized to ensure a long service life of the repair material, avoiding new repairs in a short period. The six steps defined for a good patching repair system require more detailed testing to determine if the product is consistently effective. Steps 4 and 5 require more attention regarding the adherence bridge and the durability properties of the repair material, which could be mortar, concrete, or commercial variations with Portland cement as the main component. Similarly, in the near future, there is an interest in evaluating the performance of the three different materials working together (base concrete, repair material, and the adherence bridge between them) to guarantee strength compatibility between the repaired concrete and repair mortar.

On this basis, the main objective of this investigation is to determine which repair product or system would be more appropriate for a given application considering the durability of the material as the primary decision parameter.

## 2. Materials and Methods

### 2.1. Materials

According to the repair procedure of corrosion-damaged structures, a compatible repair material (i.e., with mechanical characteristics similar to those of the original) must be used, whether mortar or concrete [14]. This implies the need to determine the physical and mechanical properties of the original and the repair materials before applying the latter. Thus, four mortars were selected for the repair system in this study: a high-performance mortar, a medium-performance mortar, and two low-performance mortars. The performance definition refers to the durability of the materials, considering the durability indices used in a previous investigation [16]. The regularly used durability indices are the following: saturated electrical resistivity (*ρ*_S_), ultrasonic pulse velocity (UPV), total void content (TVC), water capillary absorption (WCA), rapid chloride permeability (RCP), and compressive strength (*fc*).

The cement used for this investigation was Moctezuma’s CPC 30 cement (Mexican cement to achieve a 28-day 30-MPa *fc*, similar to ASTM C-155’s Type I Portland Cement; it has unknown fillers, type, and quantity and is used to decrease the clinker content; no other specifications were provided by the manufacturer because of trademark restrictions) [17]. Silica sand from a mine query with a mesh size number of 89 (0.0057 in) was used as per ASTM C-33 [18]. The cement:sand proportion by mass used to elaborate the tested mortars in this investigation was 1:2.75. The mixture proportions of the conventional mortar mixtures (N1 and N2) were made considering water/cement ratios (*w*/*c*) of 0.55 and 0.80, respectively. The theory behind the mixture design follows the guidelines ACI Committee 211 [19] and NMX-C-061-ONNCCE [20]. Table 1 shows the mixture design.

Two commercially available mortars were used in this investigation (SR-60 and MR-II). Because of trademark restrictions for both commercial products, there was no detailed material content and proportioning information given by the manufacturers. The following are the only descriptions provided for the SR-60 mortar: CPC cement type; marble quarry sand as fine aggregate; low *w*/*c* ratio (<0.35); and polycarboxylates-based water reducer in powder form and integrated into the dry mixture. The product was presented in closed plastic buckets (equivalent to 20 kg volume of water), and a bottle of tap water was provided for the mixture.

The only information given for the MR-II mortar was that it contains propylene fibers. The manufacturer did not provide the fiber size or content information. This mortar was fabricated according to the manufacturer’s specifications obtained from the product’s bag: 0.190 kg of water per kilogram of product (or 9.5 kg of water per 50-kg bag). MR-II’s physical and mechanical characteristics are listed in Table 2 (obtained from the manufacturer).

Both commercially available mortars were prepared in a mortar mixer following the procedure recommended by the manufacturers (hand mixing was not allowed as per the manufacturers’ recommendations) [21]. When the mixture was fully homogenized, it was placed in the molds.

### 2.2. Specimen Dimensions

The specimens selected for this investigation were 5 × 5 cm^2^ cubes (width × height), 5 × 10 cm^2^ cylinders (diameter × height), and 10 × 20 cm^2^ cylinders (diameter × height). All mixtures were prepared to obtain at least 15 cubes, four 5 × 10 cm^2^ cylinders (small), and nine 10 × 20 cm^2^ cylinders (large).

### 2.3. Saturated Electrical Resistivity (ρ_S_) Index

For this test, the specimens were removed from the curing chamber. Wet sponges were placed on the two ends of each specimen, and stainless steel plates were placed on either end, touching the sponges. The cables of the testing equipment were connected to the plates and the equipment. Then, the voltage passed through the two plates and the resulting ionic current was measured. The equipment voltage and current values were used to calculate the electrical resistance (Re) between the plates, that is, the resistance of the mortar specimen. The resistance was measured using a commercial resistance meter as per the Mexican standard procedure NMX-C-514-ONNCCE [22] (in kilo-ohms [kΩ]). The resulting values were multiplied by a constant dividing of a specimen’s area (*A*) by its length (*L*): 5 cm for the cubes, 1.96 cm for the small cylinders, and 3.93 cm for the large cylinders. For this test, a total of 188 specimens were subjected to mechanical compression (84 SR-60, 42 MR-II, 31 N1, and 31 N2 mortars). These were measured two to three times every week, especially at 30, 60, and 105 days. The mortar specimens were always kept in a high-humidity chamber. The specimens were retrieved from the high-humidity chamber only on the measurement dates to perform this test and the following ones.

### 2.4. UPV Index

After the *ρ*_S_ values from each specimen tested were obtained, UPV tests were performed using the same mortar specimens to determine the homogeneity of cement-based materials between specimens at different ages [23]. An emitter sends an ultrasonic pulse that travels through the material studied until it reaches the receiver sensor. The distance between one of the devices (emitter) and the other (receiver) was divided by the time taken by the wave to travel from both sensors. In this test, 123 specimens were considered (80 SR-60, 19 MR-II, 12 N1, and 12 N2 mortars). After performing the electrical resistance test, the same specimens were used for the UPV tests. The UPV was measured using a commercial UPV tester. The procedure was conducted according to the guidelines ASTM C-597 [23] and DURAR [24].

### 2.5. TVC Index

After the UPV tests, a third test was performed using the same specimens. The TVC index was obtained following the ASTM C-642 [25] standard procedure. One hundred specimens were used in this test (50 SR-60, 18 MR-II, 16 N1, and 16 N2 mortars). This method defines a specimen height of ≤5 cm, so the specimens used in this test needed additional preparation by slicing the large cylinders to approximately 5 cm slices. No slicing was required for the cube and small cylinder specimens. After drying at 323 °K (50 °C) until the specimens reached a constant mass (from 20 to 35 days), an initial measurement was taken and designated as the dry mass (*m_D_*). Then, the specimens were placed in a plastic container with high humidity and rinsed with water twice per day, and mass measurements were taken until they reached a constant mass. The final constant mass, defined as the saturated mass (*m_S_*), was recorded. The water-saturated cubes were weighed using a hydrostatic balance to measure the saturated-submerged mass (*m_SS_*). The TVC was estimated using Equation (1):

(1) $$TVC\;\left(\%\right)=\frac{100\times \left(m_{S}-m_{D}\right)}{m_{S}-m_{SS}}$$

### 2.6. WCA Index

After estimating the TVC, the same mortar specimens for a particular date were used to determine the capillary absorption of the mortar. The cubes and small cylinders were tested via the Fagerlund technique [24]. In this test, four coefficients are obtained to describe the mortar and concrete capillary absorption kinetics: water penetration resistance (*m*), capillary absorption coefficient (*k*), effective porosity (*ε*_eff_), and capillary sorption (*S*). The large cylinder specimens were cut to 5 cm high slices.

Ninety-eight specimens were evaluated (48 SR-60, 18 MR-II, 16 N1, and 16 N2 mortars) in this test. The specimens were dried at 323 K (50 °C) and <30% R.H. until a constant mass was achieved. Then, the cubes were covered with a sealing material on four of their six faces and the cylinders at their curved perimeter, leaving the top and bottom faces uncovered on both specimen shapes. The top faces were covered with a removable plastic film to avoid water absorption. When the initial dry mass was obtained with sealing material cover (*W*_0_), the specimens were placed inside a container (22 ± 4 °C and ~100% R.H.). The water level reached between 3 and 5 mm deep all the time. All the specimens were kept inside their containers, and the water level was monitored daily (avoiding water loss due to water absorption or evaporation).

Mass change vs. time of the mortar specimens was registered once a day, five days a week, during the first two months. Afterward, three measurements were recorded per week for the next three months. Finally, tests were conducted once a week until the experimental period ended.

The absorption coefficients were calculated using the following equations [24]:

(2) $$m\;\left[\sec\cdot m^{-2}\right]=tn\times z^{-2}$$

(3) $$k\;\left[\mathrm{kg}\cdot m^{-2}\times \sec^{-\frac{1}{2}}\right]=\left(W_{t}-W_{0}\right)\times A^{-1}\times t^{-1/2}$$

(4) $$\varepsilon_{\mathrm{eff}}\;\left[\%\right]=0.001\times k\times m^{-1/2}$$

(5) $$S\;\left[m\times \sec^{-1/2}\right]=m^{-1/2}$$

where *k* is the slope of the linear region of the graph *(W_t_* − *W_0_)*/*A* as a function of *t*^1/2^; *m* can be determined by calculating the time *tn* required for water to get to the top face of the probe.

### 2.7. RCP Index

The RCP technique was applied as per ASTM C-1202 [26]. Large concrete cylinders (10 × 20 cm^2^) were cut laterally (sliced), and the sections were water-saturated under the procedure of a certified laboratory for at least 24 h. The slices were assembled with two acrylic cells each containing NaCl and NaOH solutions. Constant voltage (approximately 60 V) was applied between the two acrylic cells containing metallic meshes (stainless steel) for 21,600 s (6 h), and the ionic current passing between one cell mesh to another was registered (the temperature was also measured as a safety precaution). RCP is the accumulative ionic current passing into the concrete slice in Coulombs. A total of 52 slices were used in this test (28 SR-60, 12 MR-II, 6 N1, and 6 N2 mortars).

### 2.8. Compressive Strength (fc) Index

After performing all previous nondestructive tests, the small cubes and cylinders were finally tested to determine their *fc* using the same specimens in the same order as presented in this experimental procedure, following the procedure as per ASTM C-109/C-109M [27]. This test was conducted using a Universal Servo Hydraulic Testing Machine (nominal maximum capacity of 500 kN). The loading rate was ~0.25 MPa/s. An interlaced computer automatically recorded the maximum obtained load. A total of 72 specimens were used in this destructive test (23 SR-60, 14 MR-II, 17 N1, and 18 N2 mortars).

## 3. Results and Discussion

The results of this investigation are presented as an average of at least three specimens (for all three shapes) together with the percentage variation coefficient (CV%) at three ages: 30, 60, and 105 days. All specimens were kept saturated inside a high-humidity chamber until being selected for testing at the defined testing dates. The same specimens were used to determine all the evaluated durability indices, starting with the *ρ*_S_, UPV, TVC, WCA, and finally, *fc* (the specimens were discarded after *fc* testing).

### 3.1. ρ_S_ Index

As shown in Table 3, the values are similar for the MR-II, N1, and N2 mortars, and their results are considerably different from those obtained using the SR-60 mortar.

The SR-60 mortar not only presented the best performance for this test, but also the highest CV values from the four mortars tested. This performance could be because of an apparent deficiency in specimen preparation when the dimensions are greater than 5 × 5 cm^2^, such as those of the 10 × 20 cm^2^ cylinders.

The workability of the SR-60 commercial mortar during mixing was considerably low initially, but after 900 s (15 min), the mortar was almost liquid. This was due to the addition of the water reducer based on polycarboxylates, and it remains in such a state if the mixer is running. Then, the mortar was poured into the molds without following a standardized procedure [28] because of its highly fluid form.

Until recently, the *ρ*_S_ was the most common durability test used in laboratories and in the field [3,4] because it is a quick test and could be performed using small equipment. In this investigation, the mortar performance was further analyzed by comparing the *ρ*_S_ as the main durability index with the other durability indices used (UPV, TVC, WCA, and *fc*). These correlations between the *ρ*_S_ and other indices are presented as each index’s results in the following subsections.

### 3.2. UPV Index

As shown in Table 4, the average UPV index values for N1 (3.2 km/s) and N2 (3.5 km/s) were less than that of MR-II (3.8 km/s) at >100 days of curing. The SR-60 mortars (with an average UPV index of 4.2 km/s) behaved better than the conventional mortars (N1 and N2) and other commercially available mortar (MR-II).

**Figure 1:**
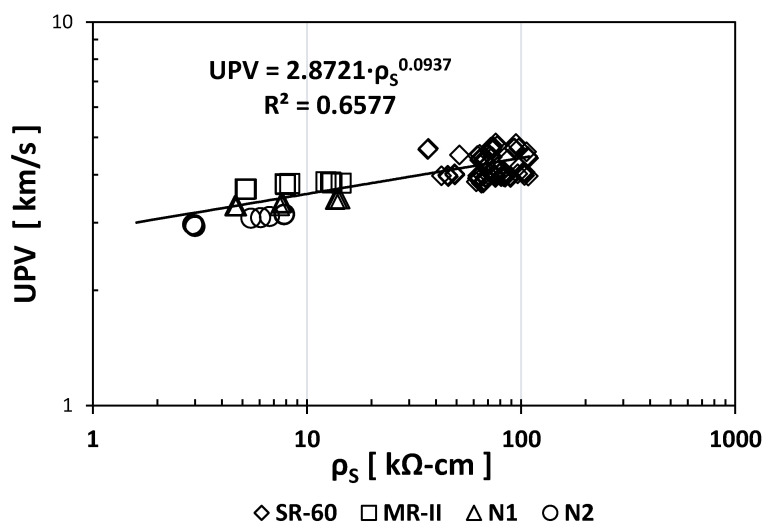
Empirical correlation between the *ρ*_S_ and UPV indices.

Similar to the *ρ*_S_ index values, the UPV index values for the SR-60 showed CV values one order of magnitude higher than those obtained for the N1, N2, and MR-II mortars. Figure 1 shows the empirical correlation between the *ρ*_S_ and UPV indices.

As shown in Figure 1, both indices did not present a strong exponent (0.0937) even though the correlation coefficient is adequate (R^2^ = 0.6577). This might be because of the small variation between the UPV index values, which change from 3.2 to 4.1 km/s (a 28% difference), compared with that of the *ρ*_S_ index values from 8 to 89 kΩ∙cm (one order of magnitude difference), giving a small exponent in the estimated correlation equation. Because the UPV index showed small variations between mixtures and thus a small exponent in the *ρ*_S_ index vs. UPV index correlation, continuing the use of the UPV index was not necessary for further empirical correlations.

### 3.3. TVC Index

As shown in Table 5, the SR-60 mortar presents an average TVC index value of just 5% compared with the results from the conventional (N1 and N2) and MR-II mortars, giving TVC index values as high as 25% for the N1 and N2 mortars and 15% for the MR-II mortar.

In Table 5, the CV values for the SR-60 mortar again showed high variability for the TVC index results obtained (between 18% and 41%), compared with those of the N1, N2, and MR-II mortars, in which the variation coefficients obtained were <11%.

**Figure 2:**
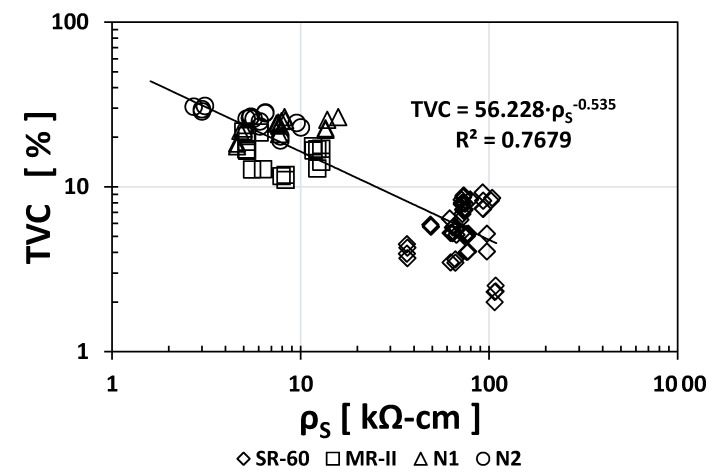
Empirical correlation between the *ρ*_S_ and TVC indices.

Figure 2 shows the empirical correlation between the *ρ*_S_ and TVC indices. The exponent in the *ρ*_S_ vs. TVC empirical correlation was −0.535, which is considerably higher than that of the *ρ*_S_ vs. UPV correlation, implying that this new correlation is stronger than the previous one. However, the N1/N2/MR-II data are separated from the SR-60 data, indicating that its continuity is not fulfilled and thus the need to obtain additional data to support this type of correlation exponent (−0.535) and coefficient (R^2^ = 0.7679).

### 3.4. WCA Index

Table 6 shows the average and the variation coefficient of the *ε*_eff_ (capillary porosity) durability index, estimated using Equation (4). Similar separations between the average *ε*_eff_ index values of the SR-60 and N1, N2, and MR-II mortars were noticeable. Up to 120 days, SR-60 presented *ε*_eff_ index values of about 3%, compared with 12, 18, and 7% for the N2, N1, and MR-II mortars, respectively.

For this index, the obtained CV values were considerably high for all the tested mortars, which might be because this index was obtained using the three specimen shapes and dimensions; thus, the specimen’s dimensions might have affected this test. The highest variation coefficients were obtained for the SR-60 mortar, which could also be because to the workability and compacting issue during the specimen fabrication.

**Figure 3:**
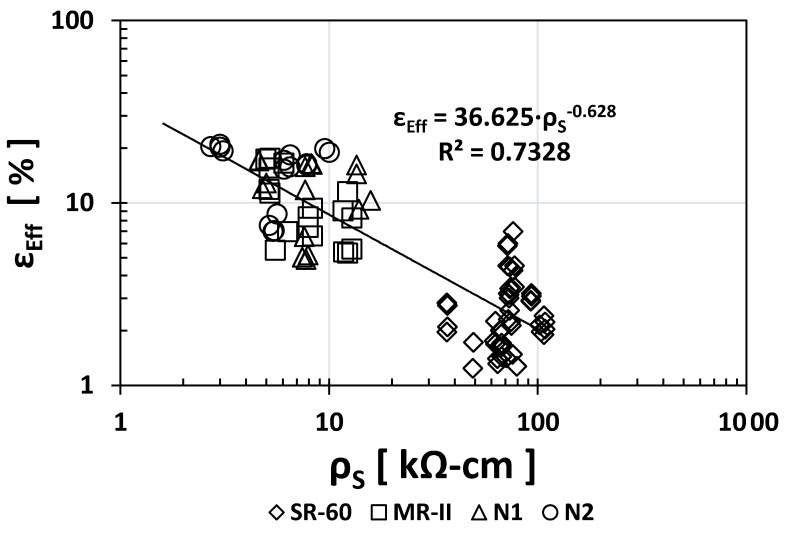
Empirical correlation between the *ρ*_S_ and ε_eff_ indices.

**Figure 4:**
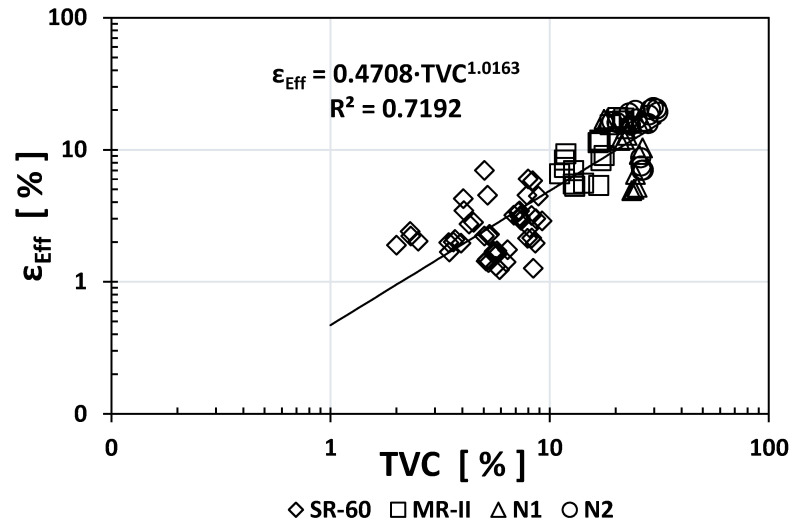
Empirical correlation between the TVC and *ε*_eff_ indices.

Figure 3 and Figure 4 show the empirical correlations between the *ρ*_S_ vs. *ε*_eff_ and TVC vs. *ε*_eff_ indices, respectively. As observed from their exponent values, both presented strong correlations: −0.628 and +1.0163. The regression coefficients are also notably high (0.7328 and 0.7192), which helped determine that these two empirical correlations are important. Only one empirical correlation (*ρ*_S_ vs. *ε*_eff_) presented a gap between the SR-60 and N1/N2/MR-II data, which needs further evaluation with additional data in the future.

Interestingly, the empirical correlation between TVC and ε_eff_ gives the form *y* = *Ax^B^*, where *A* = 0.47 and *B* = +1.02. This means that the effective capillary porosity (*ε*_eff_) is almost half the TVC and is linearly dependent because *B* is approximately equal to 1.

### 3.5. RCP Index

Table 7 shows the average and CV values for the RCP durability index. As can be observed, the N2 mortar at 60 days performed quite differently than expected. This performance might be because of specimen fabrication problems for this particular test date. The average results obtained for the same mortar return to the expected values and are similar to the average obtained at 30 days.

**Figure 5:**
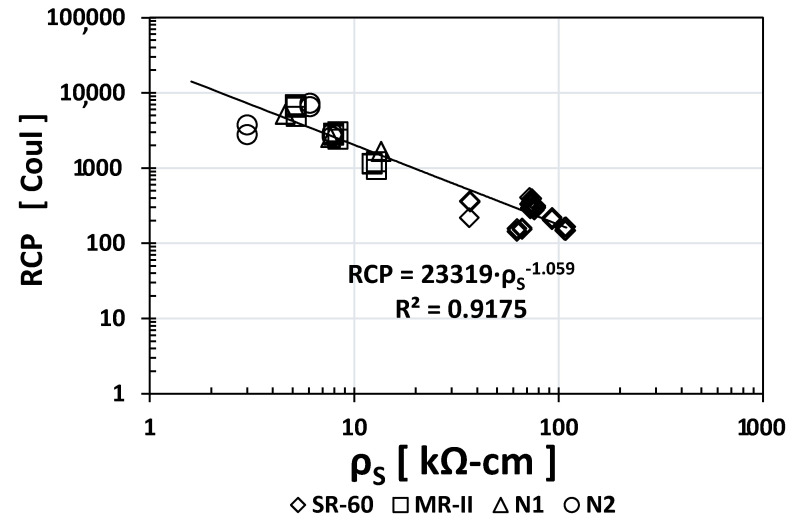
Empirical correlation between the *ρ*_S_ and RCP indices.

**Figure 6:**
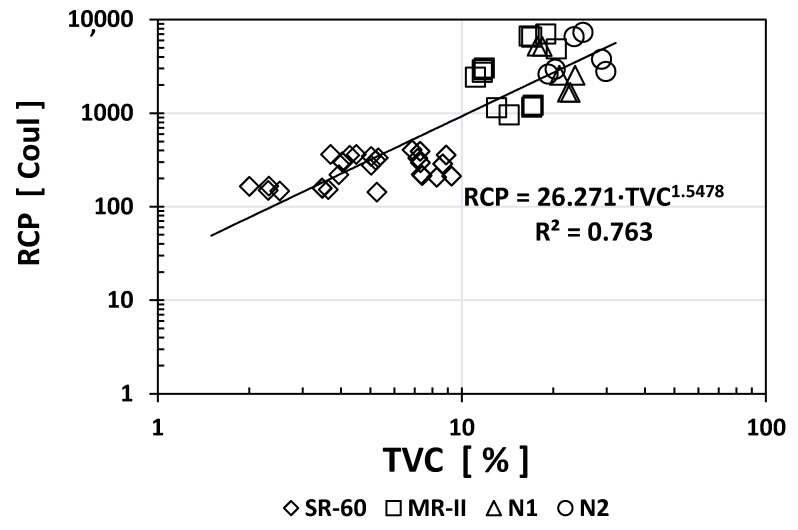
Empirical correlation between the TVC and RCP indices.

**Figure 7:**
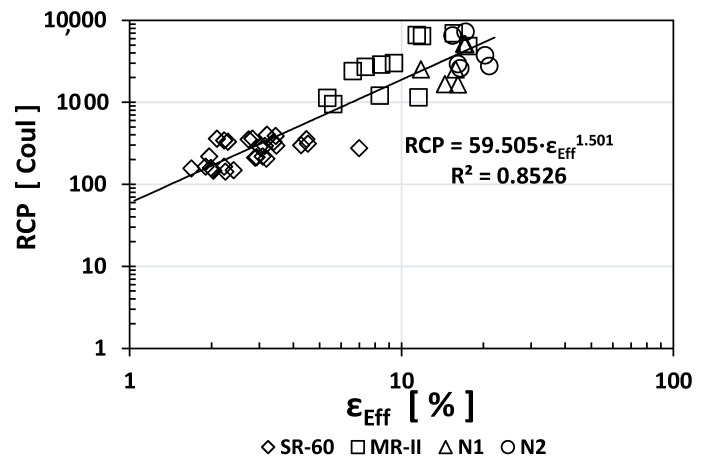
Empirical correlation between the *ε*_eff_ and RCP indices.

Similarly, to other durability indices, the RCP values obtained for SR-60 mortar were one or two orders of magnitude higher than the N1/N2/MR-II values obtained. Based on the obtained results, Figure 5, Figure 6 and Figure 7 show the empirical correlations between the RCP and the *ρ*_S_, TVC, and *ε*_eff_ indices, respectively.

As observed from all empirical correlations between the RCP index and other durability indices, strong correlations were obtained based on the “*x*” exponents (for *ρ*_S_: −1.059; for TVC: +1.5478; and for ε_eff_: +1.501) and the regression constants R^2^ (for *ρ*_S_: 0.9175; for TVC: 0.763; and for *ε*_eff_: 0.8526). Interestingly, the MR-II mortar values were located between those of the high-performance mortar (SR-60) and the low-performance mortar (N1/N2) in two of the three plotted correlations (TVC vs. RCP and *ε*_eff_ vs. RCP).

In a previous investigation, an analytical estimation of RCP as a function of *ρ*_S_ was obtained for concrete based on the geometric parameters of the test specimens and Ohms law [3,4,29]. This analytical correlation was also in the form *y* = *Ax^B^*, where *y* = RCP, *A* = 20,400, *x* = *ρ*_S_, and *B* = −1. Comparing this with the empirical correlation in Figure 5 (where *A* = 23,319 and B = −1.059), both the *A* and *B* constants for the mortar were very close to an analytical relation obtained previously [3,4,29]. This corroborates that the RCP test is, in fact, an electrical resistance test, which can be easily estimated from a *ρ*_S_ test instead, as previously recommended for either concrete or mortar [3,4,29].

### 3.6. fc Index

Table 8 shows the mortars’ mechanical performance from the average *fc* and the estimated variation coefficient values.

**Figure 8:**
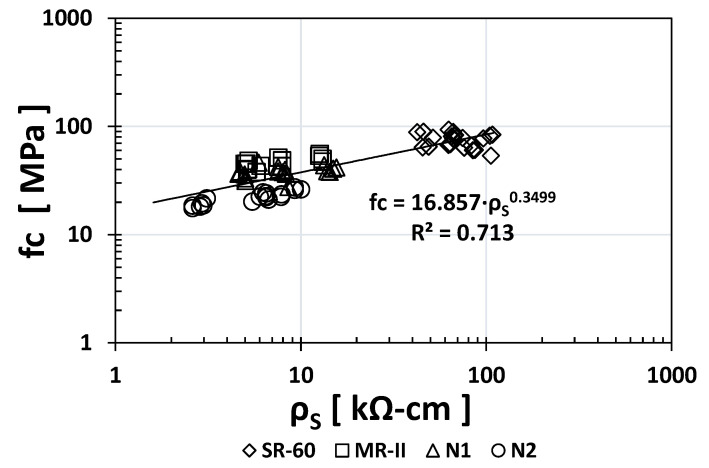
Empirical correlation between the *ρ*_S_ and *fc* indices.

**Figure 9:**
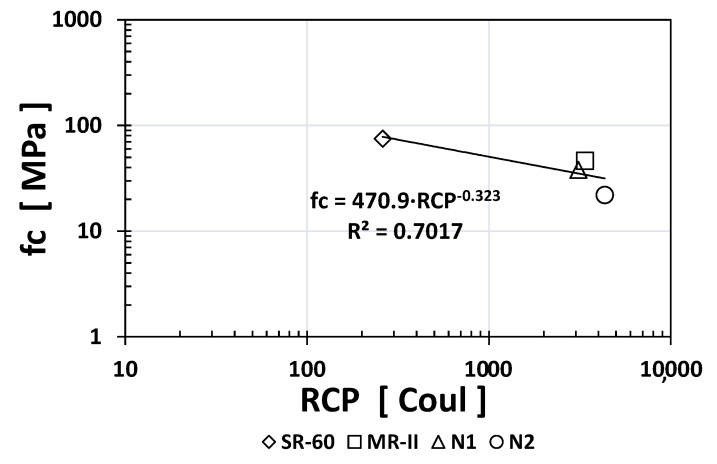
Empirical correlation between the RCP and *fc* indices.

**Figure 10:**
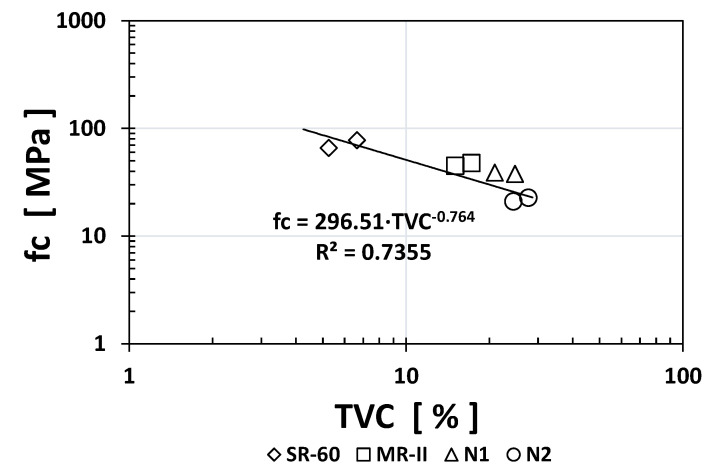
Empirical correlation between the TVC and *fc* indices.

**Figure 11:**
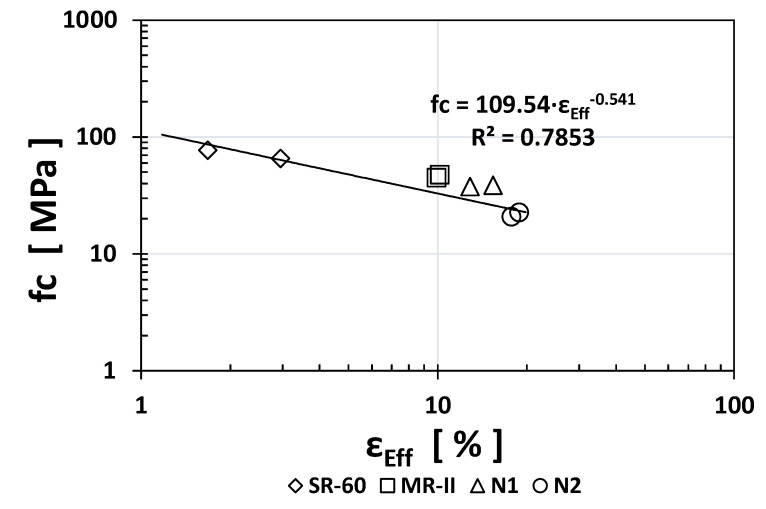
Empirical correlation between the *ε*_eff_ and *fc* indices.

Plotting the *fc* index results vs. those of all the other durability indices, Figure 8, Figure 9, Figure 10 and Figure 11 show the obtained empirical correlations. The regression coefficients were quite good, between 0.7017 and 0.7853. All *fc* index empirical correlations followed an exponential trend where the “*x*” exponent varied: +0.3499 for *ρ*_S_, −0.323 for RCP, −0.631 for TVC, and −0.457 for *ε*_eff_. These correlations could be used in the near future when additional experimental data are available to determine one index as a function of another known index.

### 3.7. Final Discussion

Based on all the experimental results obtained and the empirical correlations plotted between the evaluated durability indices, the mortars performed as expected. N1 and N2 were defined as intermediate- and low-durability mortars, respectively, based on the mechanical strength of the design used. However, the results obtained using the six performance indices exhibited that both are low-durability mortars. Conversely, the commercially available mortars MR-II and SR-60 performed in most of the tested indices as intermediate- and high-durability mortars, respectively.

The empirical correlations obtained between the evaluated durability indices show interesting direct dependences between them. This enabled performing some of them and estimating the others using the empirical equations obtained in this investigation. For example, considering that from all the indices evaluated, *ρ*_S_ is the easiest test; thus, measurements of only *ρ*_S_ would allow estimating other indices: UPV, TVC, *ε*_eff_, RCP, and *fc*.

The studied repair mortars need further evaluation based on the possible applications that may be used. Depending on the importance of the repaired element and the physical and mechanical properties of its material, the repair mortar to be chosen for this particular structural element should be adequate.

Typical repair procedures require using the repair materials with properties (physical and mechanical) similar to those of the substrate that will receive the repair material [15]. Thus, for regular concrete structures made using conventional concrete (*fc* between 20 and 30 MPa), N1 or N2 are the repair mortars that should be used.

However, these low-performance repair mortars will lead to a low service life after being placed in the repaired concrete element. Therefore, an adherence bridge (the material placed on top of the concrete under repair to enhance its adherence to the repair material) is needed to improve the performance of the entire repair system (including the repair mortar or concrete).

Based on the experimental results obtained from the tested mortars, Table 9 defines the proposed durability performance criteria for cement-based mortars. In this table, all durability indices and some value ranges defined according to the results obtained using mortars evaluated in this investigation are listed. As shown in the table, value ranges are, in some cases, similar to those defined in other standards for concrete materials [20,22,23,24,25,26], and these values are defined for cement-based mortars. These value ranges for determining mortar durability performance need to be evaluated using additional experimental data. Nevertheless, with the actual data obtained in this investigation and the empirical correlations obtained between all durability indices, excellent approximations were obtained to statistically support the ranges defined in Table 9.

## 4. Conclusions

On the basis of the experimental results obtained in this paper, the following conclusions are presented:

1. Designing conventional mortars based on the mechanical properties (*fc*) of actual standardized design methods is insufficient to obtain durable materials. Additional tests such as wet electrical resistivity (*ρ*_S_), percent TVC, effective capillary porosity (*ε*_eff_), and RPC are needed to estimate the durability indices of these cement-based materials and guarantee a durable material and, subsequently, a durable structure.
2. Excellent empirical correlations were observed from all durability indices evaluated. The *ρ*_S_ index is the most useful of the six because it can directly predict the durability performance of repair mortars using a straightforward and easy procedure. Any one of the three tests can be performed and used to extrapolate approximate experimental results for the other two indices using the proposed empirical equations. This could help engineers if a test is too difficult to perform in a short time or if there is no available equipment at the moment.
3. Evaluation criteria based on durability performance were obtained, and three performance levels for cement-based mortars were defined: low, intermediate, and high. This is important for engineering applications, because engineers could accurately predict not only the durability performance of the base concrete, but also the durability performance of the repair material with the levels defined in this investigation.

## Figures and Tables

**Table 1:** Mixture proportion for the conventional mortars tested.

Material	N1 [kg/m^3^]	N2 [kg/m^3^]
Cement	586.67	403.33
Water	322.67	322.67
Sand	957.63	1071.12

**Table 2:** MR-II’s physical and mechanical characteristics provided by the manufacturer.

Characteristics	Description	
Dry volumetric mass	1413 kg/m^3^	
Hardened volumetric mass	1690 kg/m^3^	
Compressive strength	16.0–18.0 MPa at 1 day	35.0–45.0 MPa at 28 days
Flexure strength	9.5–9.7 MPa at 1 day	11.0–12.0 MPa at 28 days
Tensile strength	3.2–4.0 MPa at 1 day	4.0–4.6 MPa at 28 days

**Table 3:** Average and coefficient of variation (CV) values for the *ρ*_S_ index. Results are presented as average (CV%).

*ρ*_S_/Type of Mortar	*ρ*_S_ [kΩ-cm] 30 d	*ρ*_S_ [kΩ-cm] 60 d	*ρ*_S_ [kΩ-cm] 105 d
N1 Average	4.95 (5.12%)	7.98 (7.96%)	13.76 (7.31%)
N2 Average	2.86 (6.04%)	6.17 (7.46%)	7.83 (11.97%)
MR-II Average	5.36 (8.76%)	6.01 (20.49%)	10.98 (4.91%)
SR-60 Average	50.59 (26.05%)	71.46 (8.56%)	88.21 (14.72%)

**Table 4:** Average and CV values for the UPV index. Results are presented as average (CV%).

UPV/Type of Mortar	UPV [km/s] 30 d	UPV [km/s] 60 d	UPV [km/s] 105 d
N1 Average	3.32 (0.15%)	3.37 (0.83%)	3.46 (0.21%)
N2 Average	2.95 (0.58%)	3.10 (0.37%)	3.15 (0.13%)
MR-II Average	3.68 (0.10%)	3.79 (0.31%)	3.83 (0.20%)
SR-60 Average	4.33 (6.94%)	4.24 (9.15%)	4.23 (7.02%)

**Table 5:** Average and CV values for the TVC index. Results are presented as average (CV%).

TVC/Type of Mortar	TVC [%] 30 d	TVC [%] 60 d	TVC [%] 105 d
N1 Average	20.14 (10.61%)	24.27 (6.41%)	24.27 (7.56%)
N2 Average	30.08 (3.06%)	26.24 (5.72%)	21.76 (9.72%)
MR-II Average	19.35 (10.53%)	12.04 (4.66%)	15.97 (10.74%)
SR-60 Average	4.39 (20.17%)	6.88 (18.71%)	5.42 (41.21%)

**Table 6:** Average and CV values for the *ε*_eff_ index. Results are presented as average (CV%).

*ε*_eff_/Type of Mortar	ε_eff_ [%] 30 d	ε_eff_ [%] 60 d	ε_eff_ [%] 105 d
N1 Average	14.75 (15.93%)	10.25 (49.18%)	12.54 (22.68%)
N2 Average	20.24 (2.91%)	12.14 (38.34%)	17.87 (9.13%)
MR-II Average	15.07 (16.77%)	5.64 (43.29%)	7.54 (30.74%)
SR-60 Average	2.05 (21.95%)	2.78 (50.08%)	2.86 (47.48%)

**Table 7:** Average and CV values for the RCP index. Results are presented as average (CV%).

RCP/Type of Mortar	RCP [C] 30 d	RCP [C] 60 d	RCP [C] 105 d
N1 Average	5140 (0.45%)	2526 (0.12%)	1660 (0.27%)
N2 Average	3274 (14.83%)	6929 (5.33%)	2779 (5.81%)
MR-II Average	6240 (13.03%)	2777 (-)	1948 (8.43%)
SR-60 Average	324 (18.71%)	279 (34.03%)	222 (26.25%)

**Table 8:** Average and CV values for the *fc* index. Results are as average (CV%).

*fc*/Type of Mortar	*fc* [MPa] 30 d	*fc* [MPa] 60 d	*fc* [MPa] 105 d
N1 Average	35 (7.15%)	39 (16.10%)	39 (4.73%)
N2 Average	19 (3.42%)	23 (7.51%)	24 (4.56%)
MR-II Average	41 (6.86%)	48 (6.39%)	32 (2.79%)
SR-60 Average	78 (13.23%)	69 (9.55%)	77 (12.93%)

**Table 9:** Defined durability performance levels.

Durability Index Test	Low Performance	Intermediate Performance	High Performance
ρ_S_, kΩ∙cm	<9	10–50	>51
UPV, km/s	<2.9	3–4	>4.1
TVC, %	>16	10–15	<9
WCA, %	>11	5–10	<4
RCP, Coulomb	>4001	1000–4000	<999
*fc*, MPa	<29	30–50	>51

## Data Availability

This is an ongoing investigation; therefore, data are not available until the investigation ends.
